# Systematic review of mathematical models exploring the epidemiological impact of future TB vaccines

**DOI:** 10.1080/21645515.2016.1205769

**Published:** 2016-07-22

**Authors:** Rebecca C. Harris, Tom Sumner, Gwenan M. Knight, Richard G. White

**Affiliations:** a TB Modelling Group, TB Centre and Centre for the Mathematical Modelling of Infectious Diseases, Faculty of Epidemiology and Population Health, London School of Hygiene & Tropical Medicine, London, UK; b National Institute for Health Research Health Protection Research Unit in Healthcare Associated Infection and Antimicrobial Resistance, Imperial College London, London, UK

**Keywords:** epidemiology, infectious disease dynamics, mathematical model, systematic review, theoretical models, tuberculosis, vaccines

## Abstract

Mathematical models are useful for assessing the potential epidemiological impact of future tuberculosis (TB) vaccines. We conducted a systematic review of mathematical models estimating the epidemiological impact of future human TB vaccines. PubMed, Embase and WHO Global Health Library were searched, 3-stage manual sifted, and citation- and reference-tracked, identifying 23 papers. An adapted quality assessment tool was developed, with a resulting median study quality score of 20/28. The literature remains divided as to whether vaccines effective pre- or post-infection would provide greatest epidemiological impact. However, all-age or adolescent/adult targeted prevention of disease vaccines achieve greater and more rapid impact than neonatal vaccines. Mass campaigns alongside routine neonatal vaccination can have profound additional impact. Economic evaluations found TB vaccines overwhelmingly cost-effective, particularly when targeted to adolescents/adults. The variability of impact by setting, age group and vaccine characteristics must be accounted for in the development and delivery of future TB vaccines.

## Introduction

Although Bacillus Calmette–Guérin (BCG) is a longstanding cornerstone of the Expanded Programme on Immunization (EPI) schedule, the only licensed vaccine against tuberculosis (TB) disease, and one of the most widely used vaccines worldwide, it provides variable protection against pulmonary forms of tuberculosis disease and an uncertain duration of protection.^1,2^ Tuberculosis is responsible for the largest number of annual deaths from a single infectious agent, estimated at 1.5 million in 2014, of which 91% were adults.^3^ Of 9.6 million incident cases, 37% were undiagnosed or unreported.^3^ Hence there remains a substantial unmet need for preventative measures such as new TB vaccines, particularly for protection against pulmonary disease in adult populations, which is clinically challenging to manage as well as being the source of most on-going transmission. It is a widely held view that new TB vaccines will be essential to the efforts to meet the World Health Organization (WHO) End TB Strategy 2035 goals and WHO 2050 goal of elimination of TB as a public health problem.^3-5^ With 15 candidates currently in clinical trials, including one in each of phase IIb and III, the current TB vaccine pipeline is the most promising to date.^3^

The field is yet to see clinical efficacy in a novel candidate and the lack of an immunological correlate of protection for TB makes identifying promising candidates challenging, therefore clinical trials are long and costly, with limited guarantee of success. Mathematical models are invaluable tools for exploring the potential epidemiological impact of different future vaccine profiles and implementation strategies. They can inform the development of target product profiles and clinical development plans leading to vaccine candidates ready for licensure in the target populations in which they would have the greatest population-level impact. Modeling results are also important in advocating for vaccine development and investment.

The TB vaccine pipeline consists of a variety of vaccine profiles. Novel TB vaccine profiles can be categorized into four dimensions: the host infection status required for efficacy (pre-, post- or, pre- and post-infection: PRI, PSI and P&PI), an effect type (prevention of infection, disease, or infection and disease: POI, POD or POI&D), an efficacy, and a duration of protection (see Table 1 for definitions and abbreviations). Maximization of vaccine efficacy and duration of protection is obviously desirable, but given the challenges faced in TB vaccine development, a partially protective vaccine with limited duration of protection is a likely outcome. Similarly, a P&PI and a POI&D vaccine would have greatest impact, but given this may not be possible, less is known about the relative advantages of efficacy with different host infection status and effect types, particularly when considering extrinsic factors such as the age-structure and temporal trends of local epidemiology and other control measures. Therefore, modeling is a logical framework for estimating the influence of such factors on the population-level impact of future vaccines. This is important for rational development of target product profiles, minimum acceptable profiles, identifying target populations for vaccination, and estimating cost effectiveness of such vaccines.

**Table 1.** Vaccine profile definitions.

Vaccine characteristic	Terminology	Definition	Abbreviation
Host infection status required for efficacy ^a^	Pre-infection	Protects when delivered to uninfected populations. Does not protect when delivered to infected or previously infected populations.	PRI
	Post-infection	Protects when delivered to latent (and/or recovered) populations. Does not protect when delivered to uninfected populations.	PSI
	Pre- and post-infection	Protects when delivered to uninfected, latently infected or recovered populations	P&PI
Effect type (infection/disease transition protected against)	Prevention of infection	Effective against the acquisition of *M.tb* infection (uninfected to infected state)	POI
	Prevention of disease	Prevents progression to active disease (uninfected or infected to disease state)	POD
	Prevention of infection and disease	Prevents both infection and development of disease	POI&D
Efficacy	Vaccine efficacy	Protection provided by the vaccine. Can be “take” or “degree”	VE
Duration of protection	Duration of protection	Time during which vaccine remains efficacious. May include waning of protection	—

a *M.tb e*xposure without infection, and immune priming with BCG or another vaccine, are not included within this definition, as exposure would not impact vaccine response, and priming could be under the control of the public health system.

As a growing field of research, publication of mathematical models assessing the potential impact of future TB vaccines has increased in recent years, yet no systematic review exists of this literature. Given the importance of this information for rational decision making in TB vaccine development and the strength of the current pipeline, a review of the literature was considered of importance. We therefore conducted a systematic review of published literature to answer the research question: what is the epidemiological impact of future human TB vaccines delivered to any age group when compared to no vaccination, other future vaccine profiles or other TB control interventions, as estimated using mathematical models? The aim was to provide a summary of the modeling methodology used, the characteristics of future TB vaccines explored using modeling, and the comparative epidemiological impact of such novel vaccine profiles.

## Methods

### PICOS framework

The PICOS framework was employed to define the review research question (Table S1). Searches were restricted to human studies. The interventions of interest were future, new, pipeline or theoretical vaccines for human tuberculosis. Papers exploring the impact of a single, defined-efficacy BCG vaccine were excluded; however, those exploring impact of a nominally BCG vaccine but with varying vaccine efficacy were considered for inclusion as these could be considered reflective of other theoretical vaccines. A broad definition was applied for the comparator of interest, therefore studies comparing new vaccines to no vaccine, BCG-only, alternative new vaccines or alternative currently available interventions were considered. Only articles reporting epidemiological outcomes, such as the impact on rates or absolute numbers of incidence, prevalence or mortality, or alternatively the number needed to vaccinate per case/death averted or cost-effectiveness measures, were included. Within-host impact models exploring immunological outcomes were excluded. The research question focused on the use of mathematical modeling as the study design. Narrative reviews and commentaries were excluded unless providing new modeling analyses or outcomes. No limits were placed upon publication dates.

### Search strategy

Three electronic healthcare sources (PubMed, Embase and WHO Global Health Library) providing access to seven databases (PubMed/Medline, Embase, African Index Medicus, LILACS, SEARO Index Medicus, WPRO Index Medicus, EMRO Index Medicus) were searched. A comprehensive search strategy was developed using the defined PICOS framework, using free text and Mesh/Emtree/DeCS terms tailored by database for groups of terms covering tuberculosis, vaccines and modeling (Table S2). All searches were run with the “human” filter, and the WHO Global Health Library search was limited to regional databases to avoid duplication. No language or publication type limits were applied. Searches were conducted on the 12th January 2016.

### Study selection and data extraction

This research was conducted in accordance with the York Centre for Reviews and Dissemination guidance for undertaking reviews in healthcare.^6^ Three-stage manual sifting of titles, abstracts, then full texts was employed by the primary reviewer to apply the pre-defined inclusion/exclusion criteria described in Table 2. Where uncertainty with regards to inclusion at full text existed, a low threshold was used to trigger assessment by a second reviewer (TS). Reasons for exclusion at the full text stage were recorded. Data were extracted from eligible papers into a piloted, standardized Excel database by a first reviewer (RH), and fully checked by a second (TS/RW). Reference lists were hand searched and onward citation searching was conducted using Web of Science for all included studies. Qualitative synthesis of extracted data was employed to produce a narrative summary of included literature.

**Table 2.** Inclusion and exclusion criteria applied in manual screening of articles.

Inclusion Criteria
Mathematical model
Systematic review of models of novel/future/hypothetical TB vaccine, or commentary adding to the analyses/interpretation of models reported elsewhere
Intervention is novel/future/hypothetical vaccine against tuberculosis or of an unspecified novel TB intervention with characteristics in-line with a vaccine
Reported outcomes are of the epidemiological impact of vaccine(s) (e.g. incidence, prevalence, mortality, number needed to vaccinate, cost effectiveness)
Exclusion Criteria
Within-host/immunological vaccine impact models
Review or commentary not adding to existing body of knowledge
TB epidemiological models not reporting impact of vaccine
TB epidemiological models reporting only interventions other than vaccines
Model only reporting on impact of BCG with single known/fixed efficacy
Disease or infection caused by *Mycobacterium bovis* or other non-*Mycobacterium tuberculosis* strain.

In accordance with PRISMA methodology, a protocol summary is registered on PROSPERO (reference: CRD42016033266) and the full protocol and PRISMA checklist are available in supplementary materials (Supplementary appendix C and Table S4).^7^

### Assessment of quality

We developed a new adapted tool for assessment of modeling study quality and risk of bias (Table S3). This built upon previously published frameworks for health related modeling and health economic modeling.^8,9^ By adding four more criteria to the Fone tool we allowed a more in-depth consideration of definition of model setting and population, appropriateness of modeling methodology and structure, fitting methodology, and reporting of conflicts of interest, which are essential for assessing the reproducibility of the model, alignment of the model and research question, and risks of bias.^8^ To improve usability, the adapted tool presented contains the questions to be considered for each of the 14 criteria and clear guidance on the rating of zero, one or two for each criterion. If a criterion was not relevant for a particular paper, a score of one was assigned so as not to unduly bias the scores in either direction. Papers were scored 0-2 on each of 14 criteria, giving a maximum score of 28 points. A quality of “low” (<14), “medium”(14-18), “high” (19-22) or “very high” (>22) was assigned based upon the overall score.

Assessment of quality was conducted by one reviewer (RCH), but with a low threshold for examining any uncertain scores by a second reviewer (TS). The second reviewer also reviewed one paper from each quality level to check for agreement with the scoring and overall assessment of quality. Due to potential conflicts of interest, one paper was also reviewed by a third reviewer (SR).

## Results

### Screening process

**Figure 1.**
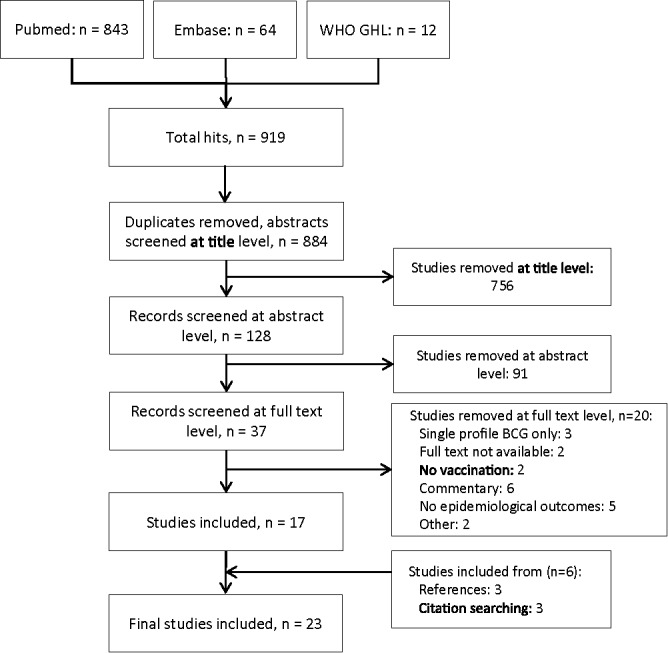
Summary of systematic screening of identified articles.

A total of 919 records, comprising 884 unique articles after removal of duplicates, were identified (Fig. 1). Title screening removed 756 articles, and abstract screening a further 91, yielding 37 articles for review at full text. Twenty articles were excluded from the review during full text screening. The two most common reasons for exclusion were commentaries providing no additional analysis (n = 6) and articles without epidemiological outcomes (n = 5). Reference and citation sear-ching identified 6 additional articles for inclusion, therefore 23 research articles were included in the review (Table 3). Although one BCG-based TB vaccine model exists from the 1960s,^10^ two models published in 1998 were the first to explore entirely novel TB vaccine profiles,^11,12^ and the majority (20/23) of included papers have been published since 2000,^4,13-31^ motivated by the promising late-stage pipeline and the push to attain challenging WHO/Stop TB global TB targets.

**Table 3.** Summary of the 23 studies included in the review.

					Vaccine Profile										
							Vaccine efficacy and coverage (%)								
Author	Year	Modeling aim	Modeling Methods	Setting	Host infection status	Effect type	Efficacy (take or degree)	Coverage	Proportion immunized ^a^(approx. % at 5 yrs)	Duration of protection (waning)	Age targeting	Infection status targeting^b^	Schedule	Time horizon (years)	Outcomes ^c^
**Quantitative outcome studies (n=18)**															
Abu-Raddad^21^	2009	Epidemiological benefits of TB	DE	SEAR (without China)	PRI	POD-f	60%(Degree)	100%	*60*%	33 yrs (0.03/yr)	Neo + Ado boost	All	Routine	35	IRR = 39%ICA = 18.2 m
					PRI	POD-fst	60%f 50%st(Degree)	100%	*60%f 50%st*	33 yrs (0.03/yr)	Neo + Ado boost	All	Routine	35	IRR = 52%ICA = 23.8 m
					PRI	POD-f	60%(Degree)	100%	*60*%	33 yrs (0.03/yr)	All	All	SM	35	IRR = 80%ICA = 68.2 m
					PSI	POD-s	50%(Degree)	100%	*50*%	33 yrs (0.03/yr)	All	L	SM	35	IRR = 37%ICA = 30.1 m
					P&PI	POD-fs	60%(Degree)	100%	*60*%	33 yrs (0.03/yr)	All	All	SM	35	IRR = 92%ICA = 80.2 m
				WPR	P&PI	POD-fs	60%(Degree)	100%	*60*%	33 yrs (0.03/yr)	All	All	SM	35	ICA = 51.5 m
				AFR	P&PI	POD-fs	60%(Degree)	100%	*60*%	33 yrs (0.03/yr)	All	All	SM	35	ICA = 47.1 m
				EMR	P&PI	POD-fs	60%(Degree)	100%	*60*%	33 yrs (0.03/yr)	All	All	SM	35	ICA = 15.4 m
				EUR	P&PI	POD-fs	60%(Degree)	100%	*60*%	33 yrs (0.03/yr)	All	All	SM	35	ICA = 10.1 m
				AMR	P&PI	POD-fs	60%(Degree)	100%	*60*%	33 yrs (0.03/yr)	All	All	SM	35	ICA = 9.1 m
Channing^22^	2014	Cost-effectiveness of adding MVA85A booster	Markov, Government perspective	South Africa	PRI	POD-d	17.3% and varied(Take)	99%	17.1% and varied	10 years	Neo BCG + 4m MVA	n/s, but likely U	Routine	10	Incremental CCA = USD 1,150Incremental CDA = USD 284,017Threshold VE = 41.3%
Cohen^14^	2008	Effect of strain diversity on vaccine performance	DE	Global (prevalence 220cases/100,000 population)	PRI PSI	POI&D-isf POD-s	Calibrated so both vaccines have equal VE against strain 1(Degree)			n/s n/s	n/s n/s	n/s n/s	n/s n/s	n/s n/s	Similar impact on quantity and distribution of strains at equilibrium. PSI demonstrated slower impact.
Ditkowsky^15^	2014	Cost-effectiveness of BCG booster	Markov, Societal perspective	South Africa	PRI	POD-f	*70-85*%,^*e*^ 0% VE in AIDS(Degree)	81% receiving prime and boost	*57-69*% ^*g*^	10 years (linear over 10 years)	Neo BCG + 4m boost	n/s	Routine	10, one cohort	Infant booster with new TB vaccine less costly than BCG alone.ICA = 2,800-4,160 (40-70%)CA = USD 7.69m - 16.68m (40-70%)
Dye^23^	2000	Impact of future vaccines on TB control	DE	n/s	PRI	POI-i	n/s(n/s)	n/s	70%	n/s	All + Neo	All	1M + routine neo	25	IRR approx. 80%
					P&PI	POD-d	n/s(n/s)	n/s	70%	n/s	Neo	All	Routine	25	IRR approx. 25%
					P&PI	POD-d	n/s(n/s)	n/s	70%	n/s	All + Neo	All	1M + routine neo	25	IRR approx. 85%
Dye^16^	2008	Assessing impact and pipeline measures for elimination	DE	World, excluding sub-Saharan Africa and HIV (1030cases/million pop)	PRI	POI-i ^h^	n/s(Take)	n/s	*0-38% of uninfected*	n/s, assumed lifetime (none)	n/s	U	Continuous	43	IR (2050) approx. 10/million/yr. Greater impact than PSI when low rates of treatment of active disease.
					PSI	POI&D-is	n/s(Take)	n/s	*0-38% of latent*	Lifetime (none)	n/s	L	Continuous	43	IR (2050) approx. 100/million/yr. Greater impact than PRI when higher rates of treatment of active disease.
					P&PI	PO&ID-is^h^	n/s(Take)	n/s	*0-38% of people*	Lifetime (none)	n/s	All	Continuous	43	IR (2050) <0.2/million/yr
Dye^24^	2013	Cost effectiveness of BCG revaccination of TST negatives in adolescence	DE	Cape town, South Africa	PRI	POI-i ^h^	n/s(Take)	n/s	10-80%	10 years (exact)	Ado, HIV negative	U	Routine	Cohort lifetime	ICA = 7.5-17% (40-80% VE over cohort lifetime)Cost/DALY averted = USD 52-4,540 (80-10% VE)
Dye^4^	2013	Prospects for elimination using various control measures	DE	Typical high burden country (1100 cases/million pop/yr, CFR 16%)	PRI	POI-i	n/s(Take)	n/s	*76% at 5 years*	n/s	All	U	Continuous	35	IR (2050) = 130/million/yr
					PSI	POI&D-is	n/s(Take)	n/s	*34% and 53% at 5 years*	Lifetime	All	L	Continuous	35	IR (2050, 14%) = 20/million/yr
				South Africa (9800 cases/million/yr)	PRI	POD-d	n/s(Take)	n/s	*70% by 2050*	Lifetime	n/s	U	Continuous	25	Similar reductions to PSI profile below
					PSI	POD-d	n/s(Take)	n/s	*75% by 2035*	Lifetime	n/s	L	Continuous	25	IR (2050) = 1,400 cases/million/yr
				China	PRI	POD	n/s(Take)	n/s	n/s	n/s	n/s	U	Continuous	25	“Limited impact”
					PSI	POD	n/s(Take)	n/s	n/s	n/s	n/s	L	Continuous	25	PSI required for elimination IR(2050) < 1 case/million/yr
				India	PRI	POD	n/s(Take)	n/s	33% by2030, 100% by	n/s	n/s	U	Continuous	25	“Modest impact” by 2050
					PSI	POD	n/s(Take)	n/s	2050	n/s	n/s	L	Continuous	25	IR (2050) < 1 case/million/yr
Knight^27^	2014	Cost effectiveness of future TB vaccines	DE	Low and middle income	P&PI	POD-d	40%, 60%, 80%, reduced by 40% (10-70%) in HIV (Take)	22–99% (country specific)^g^	*9-79*%	5yr, 10yr, lifetime (exact)	Neo	All	Routine	26	ICA (10y/60%) = 0.89mCost/DALY averted (10y/60%) = $1692Only CE in infants if vaccine 80% VE with lifelong protection
					P&PI	POD-d	40%, 60%, 80%,reduced by 40% (10-70%) in HIV (Take)	16-99% ado,68-91% adu(country specific)^j^	*6-79% ado, 27-73% adu*	5yr, 10yr, lifetime (exact)	Ado +Adu	All	Routine Ado + mass Adu (interval=duration)	26	ICA (10y/60%) = 17mCost/DALY averted (10y/60%) = $149CE threshold per dose = USD 4/20 in low/upper-middle income. All scenarios cost effective, some cost saving.
Lietman^28^	2000	Model used to assess impact of future TB vaccine	DE	Not clearly described, appears to start around incidence of 190cases/100,000pop/yr	PRI	POI&D-id	50%(Take & Degree)	88%	*44*%	Lifetime	Neo	U	Routine	40	IRR (40y) approx. 33%
					PRI	POI&D-id	50%(Take & Degree)	88%	*44*%	Lifetime	Neo + All	U	Routine +1M	40	IRR (40y) approx. 45%. Most effective of the strategies
					PRI	POI&D-id	50%(Take & Degree)	88%	*44*%	Lifetime	All	U	1M	40	IRR (40y) approx. 10%. Rebound observed as cohort age out.
					PSI	POD-s	50%(Take & Degree)	“88% … will eventually be vaccinated”	*44%, unclear timeframe*	Lifetime	All	L	SM	40	IRR (40y) approx. 45%. Fastest impact on incidence rate.
Murray^12^	1998	Impact of global control strategies	DE	Global (with 5 regional sub-models within)	PRI	POI-i	20%, 50%, 80%(Degree)	66%	*13-55*%	n/s	Appears all	U	SM	17	ICA (global)=10.5-37mICA (Asia) = 6.6-23.2mICA (SSA) = 3.4-12.2m
					P&PI	POD-f	20%, 50%, 80%(Degree)	Scale up to 80% over 10 years	*8-32*%^*k*^	n/s	Neo + All	All	Scale up mass (10 years), routine Neo after	17	ICA (global) = 16.2-51.6mICA (Asia) = 10.1-32.3mICA (SSA) = 5.4-17.1m
Pienaar^29^	2010	Effect of novel TB vaccine in hypothetical township	DE, household	Hypothetical township	PRI	POI	25%, 50%, 75%, 95% (n/s)	100%	*25-95*%	n/s, assumed lifelong	All	U	SM	10	Population infectious fraction reduced by approx. 7-70% after 120 months depending on VE
Rahman^30^	2001	Universal BCG (variable efficacy) for Japanese infants	Simple mathematical including transmission	Japan, hypothetical cohort	PRI	POD-d	40%, 60%, 80% (Take)	95%	*38-76*%	10 years	Neo	All	Routine	10, one cohort	CA = 111-542 (40-80% VE)CCA = USD 35,950-175,862 (80-40% VE)NNV =2,125-10,399 (80-40% VE)
Revelle^10^	1967	Optimization of TB control measures (variable efficacy of BCG)	DE, Optimization	Developing nation, high prevalence of active cases	PRI	POD-d	0%, 30%, 70%(Degree)	100%	*0-70*%	n/s, assume lifetime	Neo + All	U	Routine (10-13yrs) + 1M	20	Optimization of combination of vaccination and treatment
Rodrigues^18^	2009	Impact of vaccinating high risk groups versus uniform coverage	DE	‘Resemble… developed country’	PRI	POI-i	75%(Degree)	Varied to achieve epidemiological targets	Varied	n/s	Neo High risk	U U	n/s n/s	n/s n/s	Targeted vaccination better than universal only when transmission is below the reinfection threshold.^l^
Tseng^31^	2011	Cost effectiveness of novel vaccines	Markov	Zambia	PRI	POD-f	70%, 0% if AIDS (Degree)	92%	*64*%, 0% if AIDS	10 years (linear)	Neo	U	Routine	30	ICA=932CA=USD 3.6mCost saving after 1 year
					PRI	POD-f	70%, 0% if AIDS(Degree)	92%	*64*%, 0% if AIDS	10 years (linear)	Neo+ Ado boost	U	Routine	30	ICA=1863CA=USD 5.6mCost saving after 6 years, greater savings than neonatal-only ≥16 years
Young^19^	2006	Estimate the impact of novel TB vaccines	DE	South Asia (200cases/100,000 population)	PRI	POD-d	n/s (n/s)	n/s	70%	n/s	Neo	U	Routine	35	IR (2050) approx. 70/100,000/yr
					PRI	POD-d	n/s (n/s)	n/s	70%	n/s	All + Neo	U	1M+ Routine	35	IR (2050) = 20/100,000/yr
					PSI	POD-d	n/s (n/s)	n/s	70%	n/s	All	L	Unclear if SM or 1M+Routine	35	IR (2050) approx. 50/100,000/yr
					P&PI	POD-d	n/s (n/s)	n/s	70%	n/s	All	All	Unclear if SM or 1M+Routine	35	IR (2050) = 14/100,000/yr
Ziv^20^	2004	Public health impact of new TB vaccines	DE	‘High burden settings’ (100-200cases/100,000 population)	PRI	POI&D-ifs	50-90%(Degree)	60-90%	*30-81*%	10-30 years	All	U	1M + Routine U	40	ICA (10y) = 23%
						PSI	POD-s	50-90%(Degree)	60-90%	*30-81*%	10-30 years	All	L	1M + Routine L	40	ICA (10y) = 34%Impact of the 2 profiles becomes similar after 20-30 years.

					Vaccine Profile										
							Vaccine efficacy and coverage (%)								
Author	Year	Modeling aim	Modeling Methods	Setting	Host infection status	Effect type	Efficacy (take or degree)	Coverage	Proportion immunized ^a^(approx. % at 5 yrs)	Duration of protection (waning)	Age targeting	Infection status targeting^b^	Schedule	Time horizon (years)	Outcomes ^c^
**Analytical studies (n=5)**															
Bhunu^13^	2008	Effect of pre- and post- infection vaccines	DE (Analytical)	n/s	PRI	POD-f	100%(Take)	80%/yr	*100*%	(0.0002/yr)	n/s	U	n/s	200	Reduces infectious proportion approx. 75% compared to BCG-only, does not eliminate.Combined with treating latents and infectives, TTE = 15 yrs
					PSI	POD-s	75%(Degree)	80%/yr	*75*%	(0.0002/yr)	n/s	L	n/s	200	Combined with treating infectives, TTE = 20 years
Castillo-Chavez^11^	1998	Optimal age targeting of novel vaccines	DE (Analytical)	n/s	PRI	POI-i	0-100%(Degree)	Rate part of model optimization	n/s	n/s	1 vs. 2 age groups	U	n/s	n/s	Optimal age targeting schedule to minimize cost or effective reproduction number
Gomes ^17^	2004	Assess impact of ‘reinfection threshold’ on VE	DE (Analytical)	n/s	P&PI	POI-i	75-80%(Degree)	95%-100%	*71-80*%	n/s (none)	Birth	n/s, but likely U	SM	400	VE≤natural immunity=impact only seen at low prevalence (i.e. below reinfection threshold). VE > natural infection immunity =impact seen over wider range of prevalence (i.e., reinfection threshold increased).
Gomes ^25^	2007	Impact of reinfection on post-infection interventions	DE (Analytical)	n/s	PSI	POD-s	n/s(Degree)	n/s	Varies by L or recovered population	n/s	n/s	L & Recovered	SM	100	Bistable dynamics of magnitude of impact depending on whether prevalence above/below a threshold.
Hawn^26^	2014	How *M.tb* transmission affects whether VE is readily observed or masked	Statistical(Analytical)	n/s	PRI	POI-i	60%(Degree)	n/s	n/s	n/s	n/s	n/s	n/s	n/s	Fewer high-dose exposures more likely to attenuate vaccine efficacy than larger number of low dose exposures

1M: One-time mass campaign;

a Proportion immunized is the proportion of the population protected, defined as coverage times vaccine efficacy. Where not reported in the article, we have calculated this value (indicated by italics) by multiplying vaccine efficacy by coverage (or where vaccination rates are given, by estimating coverage at 5 years after vaccine introduction), though it should be noted that this does not account for waning.

Ado: Adolescent;

Adu: Adult;

b In population vaccinated, “all” refers to vaccination of all infection status populations except those with active disease.

c here large volumes of data were available, key outcomes of interest were reported for each vaccine type.

CA = Cost averted;

CCA = Cost per case averted;

CDA = Cost per death averted;

CE: cost-effective;

d : protection against progression to disease;

DA: Deaths averted;

DE: compartmental, deterministic, dynamic, difference or differential equations;

e Calculated, as BCG assumed 50% efficacious and boost VE 40-70% relative to BCG alone.

f : protection against fast progression to disease;

g Calculated proportion immunized does not account for waning, however waning at 5 years in this study will be significant, therefore calculated proportion immunized will be an overestimate.

h Vaccine leads to transition directly from uninfected to recovered with no possibility of developing disease.

i : protection against infection;

ICA: Incident cases averted;

IR: Incidence rate;

IRR: Incidence rate reduction;

j Infant coverage equivalent to DTP3 coverage, adolescent coverage equivalent to school attendance at 10yrs, mass coverage 20% below regional average of rubella campaigns.

k It was assumed that the scale up of coverage to 80% over 10 years was linear, therefore at 5 years assume 40% coverage.

l Duration of disease assumption very short (1 week).

L: latently infected;

Markov: Markov decision tree analysis;

Neo: Neonatal;

NNV = Number needed to vaccinate per case averted; n/s: not stated;

POD: prevention of disease;

POI: prevention of infection;

POI&D combined prevention of infection and disease;

PRI: Pre-infection,

PSI: Post-infection,

P&PI: combined pre- and post-infection;

*s*: protection against progression to disease from slow latent state;

SM: sustained mass;

*t*: reduction in infectiousness when vaccinated;

TTE: Time to eradication;

U: Uninfected.

### Modeling methods (n = 23)

The included studies comprised 18 deterministic, compartmental, dynamic models constructed using difference or differential equations,^4,10-14,16-21,23-25,27-29^ 3 Markov decision tree analyses,^15,22,31^ one a simple mathematical model including a fixed number of transmissions per case,^30^ and one statistical model.^26^ A subset of these papers (n = 5) present analytical solutions of models and discuss the theoretical implications of a range of factors on the impact of vaccines.^11,13,17,25,26^ These 5 papers do not provide any quantitative estimates of the epidemiological impact of novel vaccines and are not discussed further. A summary of model structures and fitting methods is provided in supplementary appendix A.

### Vaccine characteristics (n = 18)

Modeled vaccine characteristics are summarized in Table 3. An anticipated vaccine efficacy in the range of 40–80% was most commonly modeled,^10,12,15,18,20-22,27-31^ though some explored the public health impact of the extremes above^15,20,29^ or below ^10,12,22,29^ this range. Vaccine efficacy was modeled as ‘take’ (i.e. a proportion of those vaccinated are completely protected, also known as an “all-or-nothing” vaccine) in 6 studies,^4,16,22,24,27,30^ and as ‘degree’ (i.e., all vaccinees receive some protection from the vaccine, sometimes known as a ‘leaky’ vaccine) in 8 studies.^10,12,14,15,18,20,21,31^ One study modeled efficacy as a combination of take and degree.^28^ The type of vaccine efficacy was not possible to identify in three studies.^19,23,29^ Those studies explicitly modeling vaccine coverage tended to model coverage of 80–100%,^10,12,15,20-22,27-31^ though some included lower coverages.^12,20,27^ Only one study assumed coverage using data reflective of country- and age-specific access to the healthcare system.^27^ Some studies use a combined parameter (‘proportion effectively immunized’) of vaccine efficacy multiplied by coverage.^4,16,19,23,24^ Where the proportion effectively immunized was not reported, we estimated the approximate proportion at five years after vaccine implementation. The scenarios of the proportion effectively immunized spanned a wide range in most studies, ranging from a lower limit of 9–38% to upper limit of 70–95%,^10,20,22,24,27,29,30,32^ though some studies did employ lower upper limits,^4,12,15,16^ and some explored just a single scenario of proportion effectively immunized within the range 44-70%.^19,21,23,28,31^ In two studies it was not possible to estimate the proportion effectively immunized.^14,18^

Most often, duration of vaccine protection was assumed to be 10 years^15,20,22,24,27,30,31^ or lifelong,^4,16,27,28^ with some assuming alternative scenarios including 5, 30 and 33 years protection.^20,21,27,31^ Seven articles did not report the duration of protection modeled,^10,12,16,18,19,23,29^ but several appeared to use lifelong protection.^10,16,29^ Waning of protection has been modeled as either exact (all depart the vaccinated state exactly at the end of duration),^4,14,16,24,27,28,31^ or linear or exponential waning throughout the duration of protection.^15,21,31^

Many of the modeling studies explored the impact of multiple vaccine profiles assuming various effect types and/or host infection statuses required for efficacy. The post-infection (PSI) vaccines modeled assumed a prevention of disease (POD) vaccine effect,^4,12,14,19-21,28^ or prevention of infection and disease (POI&D) effect.^4,16^ Pre-infection (PRI) vaccines have been modeled assuming a prevention of infection (POI),^4,12,16,18,23,24,29^ POD,^4,10,15,19,21,22,30,31^ or POI&D vaccine effect.^14,20,28^ Combined pre- and post-infection (P&PI) vaccines have been modeled assuming a POI&D^16^ or POD^12,19,21,23,27^ vaccine effect. The most frequently explored effect types were pre-infection vaccines with prevention of disease ^4,10,15,19,21,22,30,31^ or prevention of infection ^4,12,16,18,23,24,29^ effect, and post-infection vaccines with prevention of disease effect.^4,12,14,19-21,28^

Targeting of vaccination to populations with a specific host infection status (n = 12) was common in the models identified,^4,10,12,16,18-21,24,28,29,31^ as was targeting to specific age groups (n = 12).^10,12,15,18,19,21,22,24,27,28,30,31^ Pre-infection vaccines were most frequently targeted to neonates as they were a mostly uninfected population,^10,15,18,19,21-23,28,30,31^ occasionally with an adolescent boost^21,31^ or with a one-off mass campaign to all ages.^10,19,23,28^ However, several studies included an analysis of the impact of pre-infection vaccines in adolescent,^24^ high risk,^18^ or all-ages mass vaccination campaigns or routine immunization.^12,20,21,28,29^ Post-infection vaccines have mostly been modeled as delivered to all ages,^4,19-21,28^ and in several studies age targeting is not stated but is thought to be delivered to all ages.^4,14,16^ Pre- and post-infection vaccines have been modeled as delivered to neonates,^23,27^ neonates with short term all-age mass campaigns,^12,23^ routine adolescent vaccination with adult mass campaigns,^27^ or delivered to all ages.^19,21^

### Setting and population (n = 18)

Three were global studies,^12,14,16^ two regional studies (e.g. WHO regions),^19,21^ one in low- and middle-income countries,^27^ four based upon hypothetical high burden settings,^4,10,20,28^ three were set in South Africa,^15,22,24^ one in Japan,^30^ one in Zambia,^31^ one in a hypothetical township,^29^ one in a developed country,^18^ and one where the setting was not clearly stated.^23^ Two of the afore-mentioned studies also reported on country- (China, India and South Africa) or region-level models in addition to the main model reported.^4,12^

Only two of the 18 studies consider heterogeneous social mixing patterns: one included mixing by HIV status,^12^ and one household model considers different mixing patterns during community, diurnal interactions and familial interactions at night.^29^

A small number of studies included risk groups within the modeled population. HIV status is largely neglected in TB vaccine modeling, a surprising fact given its importance as a driver of the TB epidemic in Africa and parts of Asia, but perhaps linked to this population being largely excluded from vaccine trials. One model targeted vaccination exclusively to the HIV-negative population.^24^ Only four models explicitly include HIV stratification.^12,15,27,31^ Two of the Markov decision tree models included the impact of HIV on TB natural history parameters, and assumed that the vaccine was equally efficacious in early-stage HIV infection and HIV uninfected individuals, but had zero efficacy in patients with AIDS.^15,31^ Two globally-focused dynamic models included HIV strata, one of which did not alter vaccine efficacy in the HIV stratum,^12^ and the other accounted for immunocompromise through reduced vaccine efficacy in HIV-infected patients.^27^ Two of the models with HIV strata did not explicitly include antiretroviral therapy (ART) in the model.^12,31^ The Ditkowsky study assumed 58% ART coverage which provided a 75% and 9.8% decrease in HIV-related annual mortality for those with early HIV and clinical AIDS, respectively, and a 61% reduction in the risk of progression to clinical AIDS (with associated higher risks of TB disease).^15^ In the Knight model, receipt of ART doubled life expectancy, decreased rates of progression to TB disease or death, and halved the reduction in vaccine efficacy experienced due to HIV infection.^27^ Aggressive ART scale up was assumed between 2012 and 2020, increasing from the 2009 coverage value in each country by half the difference between the 2009 value and 100%.^27^

### Epidemiological impact of future TB vaccines (n = 18)

Fourteen studies had the epidemiological impact^4,12,16,18-21,23,28,29^ and/or cost effectiveness ^15,22,27,31^ of future TB vaccine candidates as a primary focus. Three studies explored the impact of a variable efficacy BCG vaccination.^10,24,30^ One strain competition model explored impact of differential vaccine effectiveness by strain.^14^ Details of the vaccine profiles and outcomes discussed are summarized in Table 3.

#### i. Age targeting: Neonatal versus all ages, adolescents or adults

Only one study offered a clear comparison of targeting a given vaccine to neonates compared to adolescents/adults.^27^ Two studies model the impact of vaccines delivered to neonates compared to delivery to all ages.^21,28^

In the study by Knight et al., implementation of routine adolescent vaccination with periodic mass adult campaigns was found to have a much greater impact than routine neonatal vaccination with a prevention of disease vaccine in low- and middle-income countries across the 2024-50 time horizon.^27^ For example, a 60% efficacious vaccine providing 10 years protection could prevent 17 million (range 11–24m) cases between 2024-2050 when delivered to adolescent/adults compared to just 0.89 million (range 0.42–1.58m) when delivered to neonates.^27^ The vaccine coverage differed in each age targeting scenario, based upon data from current vaccination campaigns relevant to those populations and so might be considered a more realistic reflection of achievable coverage. There are likely greater cost and logistical implications of delivering an adolescent/adult vaccine than a routine neonatal vaccine, however in this study it was found that due to the greater impact achieved when vaccinating older age groups, all of the vaccine profiles explored in this age group had a cost effective price per vaccine dose, whereas for neonatal vaccination some of the shorter durations of protection and lower vaccine efficacies were not considered cost effective. It was noted in the article that some conclusions may be timeframe dependent, as those receiving neonatal vaccination with long durations of protection would only just be reaching the age of high TB risk in 2050.

As would be expected, vaccinating all ages had a greater impact (80% incidence rate reduction over a 35-year time horizon) compared to vaccinating neonates with an adolescent boost (39% incidence rate reduction) with a POD vaccine in the Abu-Raddad study.^21^ Though it should be noted that the number vaccinated would be much greater in the all-ages scenario. In the other neonatal vs. all-ages study,^28^ routine neonatal vaccination was compared to a one-off mass campaign for uninfected individuals of all ages with a POI&D vaccine. In this study, mass vaccination of all ages initially provides greater epidemiological impact than neonatal vaccination. As the vaccinated cohort ages out the incidence rates rebound, whereas with sustained routine neonatal vaccination incidence rates continue to decline, and provide greater impact than a one-off mass campaign after approximately 20 years.^28^

No studies were found comparing vaccinating neonates to all ages or adults with prevention of infection vaccines, or of combining such age targeting with targeting of post-infection populations.

#### ii. Addition of mass campaigns or boosters to routine neonatal vaccination

Three studies found that the addition of one-off mass vaccination campaigns on top of neonatal routine vaccination could have profound effects on population-level impact.^19,23,28^ In a South-East Asian study exploring impact of a pre-infection prevention of disease vaccine over a 35 year time horizon, an incidence rate reduction of approximately 65% was predicted with a neonatal vaccine, compared to >90% when adding a one-off mass campaign at launch.^19^ In another study, addition of a one-off mass vaccination campaign to the routine neonatal program (with 70% of the population protected in each group) increased the reduction in incidence rate from around a quarter to approximately 85% over 25 years.^23^ In the Lietman study, addition of a one-off mass campaign to routine neonatal vaccination greatly increased the short-term impact of the program.^28^

One study assessed the impact of an adolescent booster in addition to neonatal vaccination, which in a Markov model was found to double the number of cases averted compared to neonatal vaccination alone.^31^

#### iii. Host infection status required for efficacy: Pre-infection, post-infection or pre- and post-infection

Eight studies report a comparison of vaccines effective in different host infection statuses,^4,12,14,16,19-21,28^ of which six report clear comparisons between vaccines effective pre- versus post-infection.^4,16,19-21,28^

Exploring the impact on disease incidence, three studies suggest post-infection vaccines would have the greatest impact,^4,20,28^ two suggest pre-infection would provide greatest impact,^19,21^ and one suggests that either could have greater impact, dependent upon the rate of treatment of active disease.^16^ The six studies allowing comparison are described below.

##### iii.a. Post-infection vaccines leading to greater impact on disease incidence (n = 3)

The Ziv, Lietman and Dye (2013) models report post-infection vaccines as providing greater impact on disease incidence than pre-infection vaccines.^4,20,28^ Ziv et al., compared the effect of POD vaccines targeted to uninfected (pre-infection) or infected (post-infection) populations in a hypothetical high burden setting with 28-50% latently infected and 40-60% of active cases treated and cured, and reported that post-infection vaccines would have a greater and more rapid effect on cumulative number of incident cases than pre-infection vaccines.^20^ After 10 years, post-infection vaccines prevented 34% of cumulative cases compared to 23% by pre-infection vaccines.^20^ Given 28-50% of the population were latently infected, the number vaccinated with the post-infection vaccine will be lower in most scenarios, therefore greater impact on overall disease incidence is achieved despite being given to fewer individuals. Age targeting, vaccine efficacy and coverage of target population were identical between scenarios and both were assumed to prevent development of disease, though in the pre-infection scenario the vaccine was additionally assumed to have POI activity, and therefore was a POI&D vaccine.

Similarly, in the Lietman study the pre-infection vaccine was POI&D whereas the post-infection vaccine was POD-only, but again, when comparing otherwise identical vaccines, post-infection vaccination provided the largest and fastest impact on the disease incidence rate.^28^ In a background of 50% successful cure of active disease, comparing 88% coverage of latents with a post-infection vaccine, to vaccinating 88% of newborns with a pre-infection vaccine, impact was observed more rapidly with the post-infection vaccine, and 60 years later the disease rates with a post-infection vaccine were still lower.^28^ The sizes of the latent and newborn populations are not reported, but they are unlikely to be equal, therefore the number of people effectively vaccinated and therefore the number needed to vaccinate per case averted in each of these scenarios could be very different. Even if routine neonatal pre-infection vaccination was preceded by a one-off mass campaign, the initial impact was still not as large as the post-infection vaccine, but in this scenario after 40 years the overall incidence rate reduction was similar.

In a study by Dye et al., in a “typical high burden country” with 110 cases per 100,000 population per year and 65% case detection, of which 70% are cured, effective immunization of 25%/year of the uninfected population (pre-infection) with a POI vaccine reduced disease incidence rates to 130/million population/year, whereas as little as 14%/year effective immunization of latents (post-infection) with a POD vaccine reduced the incidence much further to 20/million population/year.^4^

In all three studies reporting greater impact of post infection vaccines, the proportion assumed effectively immunized was lower in the post-infection vaccine target group.^4,20,28^ Therefore, even with lower coverage, post-infection achieved greater impact than pre-infection vaccines.

##### iii.b. Pre-infection vaccines leading to greater impact on disease incidence (n = 2)

In the Abu-Raddad and Young models,^19,21^ pre-infection vaccines demonstrated greater impact on disease incidence than post-infection vaccines. In the Abu-Raddad model, campaigns targeted at latently infected individuals (post-infection) compared to mass vaccination campaigns using pre-infection or P&PI vaccines, predicted incidence rate reductions of 37%, 80% and 92% over a 35 year time horizon, respectively.^21^ The Young study was also a South Asian model, which compared the impact of otherwise-identical PRI, PSI and P&PI vaccines implemented in 2015.^19^ The model estimated an incidence rate of approximately 20, 50, and 14 per 100,000 population per year in 2050, indicating that the most effective vaccine in this scenario would also be a pre-infection or P&PI vaccine.^19^ Though it should be noted that there was some ambiguity in the article as to the vaccine schedule, therefore there may be confounding due to differences in schedules modeled. Both the Abu-Raddad and Young models assumed that the pre-infection vaccines were prevention of disease vaccines, so would not have had a direct effect on infection.

##### iii.c. Research indicating a possible reason for such contrasting results (n = 1)

A ‘global’ study excluding sub-Saharan Africa found that at low active disease treatment rates pre-infection vaccines providing lifetime protection had greater impact on disease incidence rates than equivalent targeting of post-infection vaccines.^16^ However, when treatment rates of active disease were increased, reducing the proportion of disease from recent infection compared to reactivation, the post-infection vaccine was reported to have greater impact on disease incidence. We discuss this more below.

##### iii.d. Other factors influencing comparison of impact of pre- or post- infection vaccines

Although the proportions effectively immunized (coverage multiplied by vaccine efficacy) did not differ markedly between studies favoring pre- or post-infection vaccines, the proportion of the population latently infected will co-determine the number of people that would receive either a pre- or post-infection vaccine, and could be an important cause of differences. However, only two of these six studies reported the assumed infection prevalence.^16,20^

Time horizon could be important for this comparison. As demonstrated in the Ziv and Lietman models,^20,28^ even though post-infection vaccines may have greater initial impact, the impact of the vaccine types sometimes converge over longer time horizons due to the greater time lag before impact is observed with pre-infection vaccines. However, all six studies explore outcomes over a narrow horizon of 35-43 years and therefore between-study differences in the impact of pre- vs. post-infection vaccines do not appear attributable to time horizon.^4,16,19-21,28^ Durations of protection, and effect type for post-infection or pre-infection vaccines all vary between models, but were also somewhat similar in both groups.

#### iv. Disease stage protected against: POI, POD or POI&D

Dye et al. (2000) compared POI and POD vaccines with 70% effective immunization in a mass campaign followed by neonatal vaccination. In this study, the incidence rate reduction was greater with the POD vaccine than the POI vaccine over a 25 year time horizon.^23^ In the Murray et al. global model, sustained mass vaccination with 66% coverage of uninfected individuals for a POI vaccine prevented fewer cases (10.5m-37.0m cases averted with 20-80% vaccine efficacy over a 17 year time horizon) than a POD vaccine with mass campaigns scaling up to 80% coverage over 10 years followed by annual neonatal vaccination (16.2m-51.6m cases averted); a trend also reflected in the regional estimates presented.^12^ However, it must be noted that there are difficulties in identifying whether these are equivalent comparisons given the differences in schedule, size and infection status of population receiving vaccine and coverage.

Several models explored POI or POD vaccines compared to a POI&D vaccine. As would be expected since it is a combination of mechanisms, a number of the studies found the POI&D vaccine to be most effective.^4,14^ Those finding more impact on disease with the POD vaccine than the POI&D vaccine were likely confounded by favorable targeting of the vaccines to latently infected individuals in the POD scenario.^20,28^

#### v. Time horizon of impact

The WHO/Stop TB global targets aim to reduce TB incidence rates to 10 cases per 100,000 population per year by 2035, and less than 1 case per million population per year by 2050, termed as tuberculosis elimination.^3,5^ Several of the studies explored the potential contribution of future TB vaccines to achieving the Stop TB 2050 target, but all were published prior to release of the WHO End TB strategy containing the 2035 goals, so none directly address these targets. In two papers by Dye et al., it was shown that pre-infection POI vaccines to interrupt transmission were unlikely to reach elimination, as even with complete transmission interruption in 2015 an incidence of >100/million/year would still be expected by 2050.^4,16^ In the Dye model, a post-infection vaccine giving full and permanent protection to 14% of the latently infected population per year was estimated to reduce incidence to 20/million/year by 2050, and if combined with treatment of active disease, or a pre-infection vaccine, would achieve elimination by 2050.^4^ In country-specific models, results suggested that a post-infection vaccine (or mass preventative therapy) could achieve elimination targets in China and India.^4^ These models suggest that a post-infection vaccine, or either vaccine effect type combined with treatment of active cases, may be capable of reaching the WHO 2050 elimination goals.^4^ However, similar models for South Africa and the USA suggested that elimination was not considered feasible in these settings with novel vaccines.^4^ Results from both the Young and Knight models indicated that none of the scenarios explored would achieve incidence rates lower than around 10-20/100,000/year by 2050.^19,27^

#### vi. Settings

There was clear variability in vaccine epidemiological impact by setting. Knight et al. demonstrated a larger proportion and absolute number of cases would be averted by vaccines in low-income countries than middle-income countries.^27^ In Japan, 95% coverage of a BCG-like vaccine with 40-80% vaccine efficacy was considered not cost effective as it could only avert 10-47 cases per 100,000 children vaccinated over a 10-year horizon.^30^ Whereas with 92% coverage of a 70% POD vaccine in the Zambian setting, cases averted were much higher at 199 cases per 100,000 vaccinated at birth;^31^ though it should be noted that the Zambian model analyzed a longer 30-year time horizon. Three models explored the cost effectiveness of adding booster vaccines in South Africa, but outcomes are not comparable with the Japanese and Zambian models.^15,22,24^ In two regional models, for each of the vaccine profiles explored the greatest numbers of cases and deaths avoided were in Asia/Western Pacific, followed by sub-Saharan Africa (see Table 3).^12,21^ Much lower absolute numbers of cases were avoidable in the other regions, with the next largest impact observed in the Eastern Mediterranean region where cases averted were around a third of those in sub-Saharan Africa.^21^

#### vii. Economic models

A small number of published studies evaluated the economics of future TB vaccines (n = 7/18),^10,15,22,24,27,30,31^ with the majority (n = 6/7) evaluating cost effectiveness of new vaccines (either future or BCG based) using either static,^15,22,30,31^ or dynamic models.^24,27^ Optimization techniques were also used to consider costs associated with different control schemes.^10^ With the exception of one model exploring a very low efficacy vaccine and one exploring infant vaccination in a low burden setting,^22,30^ those evaluating cost effectiveness found new TB vaccines to be an overwhelmingly cost effective intervention, whether from the health system^24,27^ or societal perspective.^15,27,31^ More than half of the models included societal costs, highlighting that much of the economic burden of TB disease falls on the TB patient due to the long durations of treatment. The populations considered in these economic models were either theoretical^10^ or setting specific (South Africa,^15,22,24^ Zambia^31^ or Japan^30^) with only one model considering multiple settings (low- and middle-income countries).^27^ Cost effectiveness of vaccines was highly dependent on vaccine characteristics such as efficacy (with lower efficacy linked to higher costs),^7,15,22,24,30^ setting-specific burden of disease,^30^ and economic considerations such as discount rate,^22,30,31^ and time horizon.^31^

It is important to know the minimum acceptable vaccine efficacy for designing clinical trials and when making implementation decisions. Two South African Markov model threshold analyses of new vaccine boosters demonstrated that the booster strategy would be more effective, and either cost neutral or more expensive if the booster vaccine demonstrated a vaccine efficacy of approximately 40% or above.^15,22^ The Knight et al. study provided contour plots demonstrating the vaccine efficacies and durations of protection for which the vaccines would have an above-zero price at which the vaccine would be cost effective.^27^ All vaccines in the ranges vaccine efficacy 20-100% and duration of 5 years to lifelong were cost effective, and even cost saving above 20% vaccine efficacy and 10 years duration, when delivered to adolescents and adults.^27^ However, the neonatal vaccination program was only cost effective at higher values of these vaccine characteristics, with no cost effective vaccine price for a region of the plane where vaccine efficacy and/or duration were relatively low.

### Studies identified post-hoc (n = 2)

For completeness, one unpublished study and one study published after the review search date were identified. The study published in March 2016 explores the impact of spatial ‘hotspot’ targeting compared to random allocation of an adult POD vaccine in Gujarat, India.^33^ This model did not compare vaccine effect types or host infection status or age targeting, but is the first to explore spatial targeting of vaccines, and suggests spatial targeting could increase impact by 17% in the base case scenario in this setting.^33^

The unpublished study is an exploration of the epidemiological impact of different future TB vaccine profiles when targeted to adolescents (15 year olds) compared to older adults (60 year olds) in China.^34^ This model is a dynamic transmission model incorporating heterogeneous social mixing patterns by age, age-specific natural history parameters, and fitted to age-stratified epidemiological data, and will contribute to the literature on vaccine age targeting and impact of pre- versus post-infection vaccines.

### Quality assessment (n = 23)

Using the adapted quality assessment tool, scores ranged from 11 to 25 out of 28 (Table 4). Two were considered low quality,^19,23^ eight of medium quality,^4,13,17,18,25,26,28,29^ six high quality,^10,11,14,16,20,30^ and seven of very high quality.^4,12,15,21,22,27,31^ The median score was 20/28, equivalent to high quality. As the tool was not perfectly suited to analytical papers, the sensitivity of the median score was assessed by excluding analytical papers, and the overall score did not change. The major gaps observed were definitions of the population and intervention, fitting methodology, uncertainty and sensitivity analyses, data sources, and conflicts of interest and funding. Comprehensive uncertainty and sensitivity analyses were lacking in many studies. Further discussion is included in the supplementary appendix B.

**Table 4.** Quality assessment of included modeling studies.

Author	Year	Aims and objectives	Setting and population	Intervention/comparators	Outcome measures	Model structure and time horizon	Modeling methods	Parameters, ranges and data sources	Assumptions explicit and justified	Quality of data and uncertainty and/or sensitivity analyses	Method of fitting	Model validation	Presentation of results and uncertainty	Interpretation and discussion of results	Funding source and conflicts of interest	Final Score (/28)	Rating
Abu-Raddad	2009	Yes	Yes	Yes	Yes	Yes	Yes	Yes	Yes	Partial	Yes	No	Partial	Yes	Partial	23	Very high
Bhunu	2008	Yes	No	Partial	Yes	Yes	Yes	Partial	Partial	No	Partial	No	Partial	Yes	Partial	16	Medium
Castillo-Chavez	1998	Yes	Partial	Partial	Yes	Yes	Yes	Partial	Partial	Partial	Partial	Yes	Yes	Partial	Partial	20	High ^*^
Channing	2014	Yes	Yes	Yes	Yes	Yes	Yes	Yes	Yes	Yes	Partial	No	Yes	Partial	Yes	24	Very high
Cohen	2008	Yes	Partial	Partial	Yes	Partial	Yes	Partial	Yes	Yes	Partial	No	Partial	Partial	Yes	19	High
Ditkowsky	2014	Yes	Yes	Yes	Yes	Yes	Yes	Yes	Yes	Yes	Partial	No	Yes	Yes	Yes	25	Very high
Dye	2000	Partial	Partial	Partial	Partial	Partial	Partial	Partial	Partial	No	Partial	No	Partial	Partial	No	11	Low
Dye	2008	Yes	Yes	Partial	Yes	Partial	Yes	Partial	Partial	Yes	Yes	No	Partial	Yes	Partial	20	High
Dye	2013a	Yes	Yes	Yes	Yes	Partial	Partial	Yes	Yes	Yes	Yes	No	Yes	Yes	Partial	23	Very high
Dye	2013b	Yes	Yes	Partial	Yes	Partial	Partial	Partial	Partial	Partial	Yes	No	Partial	Partial	Yes	18	Medium
Gomes	2004	Yes	Partial	Partial	Partial	Partial	Yes	Partial	Yes	Partial	Partial	No	Partial	Yes	Partial	17	Medium^*^
Gomes	2007	Yes	Partial	Partial	Partial	Partial	Yes	Yes	Yes	Partial	Partial	No	Partial	Partial	Partial	17	Medium^*^
Hawn	2014	Yes	Partial	Partial	Yes	Partial	Partial	Partial	Partial	Partial	Yes	No	Yes	Partial	Yes	18	Medium^*^
Knight	2014	Yes	Yes	Yes	Yes	Yes	Yes	Yes	Partial	Yes	Yes	No	Yes	Yes	Yes	25	Very high
Lietman	2000	Yes	No	Yes	Yes	Partial	Yes	No	Partial	No	No	No	Partial	Yes	Partial	14	Medium
Murray	1998	Yes	Yes	Yes	Yes	Yes	Yes	Yes	Yes	Partial	Yes	No	Yes	Yes	No	23	Very High
Pienaar	2010	Yes	Yes	Partial	Yes	Partial	Yes	Yes	Yes	No	Partial	No	Partial	Partial	Partial	18	Medium
Rahman	2001	Yes	Yes	Yes	Yes	Yes	Partial	Yes	Partial	Yes	Partial	No	Yes	Yes	No	21	High
Revelle	1967	Yes	Partial	Partial	Yes	Yes	Yes	Yes	Yes	Partial	Partial	No	Yes	Partial	Partial	20	High
Rodrigues	2009	Yes	Partial	Partial	Yes	Partial	Yes	Partial	Yes	Partial	Partial	No	Partial	Yes	Partial	18	Medium^*^
Tseng	2011	Yes	Yes	Yes	Yes	Yes	Yes	Yes	Partial	Yes	Partial	No	Yes	Yes	Yes	24	Very high
Young	2006	Yes	Partial	Partial	Yes	Partial	Partial	Partial	Partial	No	No	No	Partial	Partial	No	12	Low
Ziv	2004	Yes	Partial	Yes	Yes	Yes	Partial	Partial	Yes	Yes	No	No	Yes	Partial	Yes	20	High
Median score		2	1	1	2	1	2	1	2	1	1	0	1	2	1	20	High

* Analytical modeling papers.

## Discussion

Modeling the epidemiological impact of future TB vaccines is a relatively young but growing field, used to inform rational decision making in portfolio strategy, prioritization of resources, and global target setting. To date, the literature remains divided as to whether vaccines effective pre-infection or post-infection would provide the greatest epidemiological impact. However, all-age or adolescent/adult targeted prevention of disease vaccines achieve greater and more rapid epidemiological impact than neonatal vaccines. Mass campaigns or boosters added to routine neonatal vaccination can have profound additional epidemiological impact. With the exception of one very low efficacy vaccine and one low burden setting, economic evaluations found new TB vaccines to be overwhelmingly cost effective, particularly when targeted to adolescent/adult age groups. The variability of impact by setting, age group vaccinated, vaccine characteristics and time frame, must be taken into account in the development and delivery of future vaccines.

Given the importance of indirect effects in this research question, the majority of the 23 included studies captured transmission through development of dynamic models. Some potential geographical bias was observed, as several either excluded sub-Saharan Africa or were based upon an Asia-like epidemic. Although the avoidable burden of disease may be higher in Asia, the high disease rates and slower progress toward global targets in the African region may mean that this region would be in greatest need of new interventions such as vaccines to meet the WHO 2035 and 2050 targets.^3^ Epidemiological outcomes were indicative of considerable heterogeneity by setting, highlighting the importance of setting-specific modeling for decision making. Only four of the models incorporated HIV strata,^12,15,27,31^ yet globally 12% of all TB cases are HIV co-infected, of which three-quarters are found in the African Region.^3^ It is currently unknown what effect HIV-infection may have on vaccine efficacy, but it is generally thought that it may be lower in this population. Therefore, models without HIV structure may overestimate vaccine impact, and models including this structure are a useful tool to conduct sensitivity analyses around HIV-related assumptions.

Historically, novel TB vaccine development has focused primarily on vaccination in infancy. However, more recently a shift in thinking has led to the prioritization of older age groups in clinical trials and development plans. For prevention of disease vaccines, this is supported by the modeling literature, which demonstrates that all-age or adolescent/adult targeted POD vaccines achieve greater and more rapid epidemiological impact than neonatal vaccines. This is epidemiologically consistent given that in many settings adults comprise the majority of the disease burden and their primarily pulmonary disease is a greater source of *M.tb* transmission than the extra-pulmonary disease prevalent in children,^3^ therefore delivery of POD vaccines directly to the adult population provides relatively immediate impact, whereas there is usually a lag of 10-20 years before any impact of long (>10 years) duration of protection neonatal vaccination can be seen.

From an implementation perspective, neonatal and adolescent vaccines could potentially be incorporated into existing delivery platforms, but developing a platform for delivery of adult vaccination could have serious resource implications. Although this is an important consideration, one model has demonstrated that adolescent/adult vaccine targeting would be more cost effective than neonatal vaccination with a POD vaccine.^27^ Alternatively, several models suggest that addition of one-off mass campaigns for all ages at initiation of routine neonatal vaccination could have a profound effect on the population-level impact.^19,23,28^ No models were identified comparing age targeted POI vaccines or combining age targeting with targeting to post-infection populations. It was also noticed by the authors that none of the models explored targeted vaccination of older adults or the elderly, which is surprising given that several high burden countries are undergoing population aging (e.g., China, Indonesia) and the higher risk of developing active disease in this age group.

Our finding that the modeling literature was equivocal as to whether post-infection^4,20,28^ or pre-infection^19,35^ vaccines would have greatest epidemiological impact is interesting and may have important implications. Given the complexity of the studies, it is not possible to confidently identify the study differences that explain these diverse findings. However, there are 2 factors that we believe are likely to be most important. Firstly, the underlying epidemiology of the population and, in particular, factors affecting the proportion of disease due to primary disease and rapid progression versus reactivation or relapse, are likely to be influential. This was best illustrated in Dye and Williams,^16^ in this study the authors increased rates of treatment of active disease, which resulted in a switch from greater impact from a pre-infection vaccine to greater impact from a post-infection vaccine (Dye and Williams, figure 6 panels a and b – by comparing new TB cases per million in 2050 in panel a versus b at increasing treatment rates per TB case). Increasing the treatment of active disease reduced transmission and the proportion of disease arising from primary infection (versus reactivation). We recreated this by coding up this model (not shown) showing that, for the same overall disease burden, when a greater proportion of disease was due to reactivation the post-infection vaccines were predicted to have the greater impact across the full range of rates of treatment of active disease explored. Secondly, the proportion of the population latently infected will co-determine the number of people that would benefit from either a pre- or post-infection vaccine. However, only two of these six models report the infection prevalence.^16,20^ Another factor that could help explain this difference is that most of the six models compared assume no relapse from the recovered class, but in the two models assuming relapse,^4,19^ it is possible that relapse rates and assumptions as to whether this group is protected by post-infection vaccination, could affect the relative impact of pre- versus post-infection vaccines. Further, although no major differences were identified here, time horizon, vaccine efficacy, and duration of protection assumptions could also potentially influence pre- vs. post-infection outcomes. Given the difficulties we have had in identifying the reasons why these models are making conflicting predictions, there is a clear need that these assumptions are reported carefully in future vaccine modeling studies, and if this question is important for vaccine development planning, there may be a need for a controlled modeling study focusing on this question.

From the vaccine development perspective, although a vaccine effective both pre- and post-infection would be the ideal scenario, pipeline candidates with either pre- or post-infection efficacy could have value, and modeling could be used to assess their relative value in different settings. Further, if a candidate could potentially exhibit different efficacy in infected versus uninfected populations, efficacy estimates from trials could be confounded by the balance between primary and reactivation disease in the trial setting. Or worse, if the recruited population is limited to either infected or uninfected individuals it is conceivable that a candidate's development could be discontinued due to poor efficacy in one population without knowing whether the candidate would have shown better efficacy in the other group. Therefore, if proven safe and immunogenic in both infected and uninfected populations, ideally both should be recruited into clinical trials and the study powered to estimate vaccine efficacy separately for those IGRA or TST positive versus negative at recruitment to improve generalizability to other settings. However, this could make trials infeasibly large, in which case enrolling both uninfected and infected populations, but powering the trial on the primary endpoint in one population, and looking for trend, safety and immunogenicity in the other population as secondary endpoints may be preferable to using a combined endpoint.

Upon implementation, sustained campaigns specifically targeting either uninfected (with a pre-infection vaccine) or latently infected populations (with a post-infection vaccine) may not be feasible. To identify such populations, TST or IGRA testing would be required. Such tests come with cost and organizational implications, and neither are perfect tests for latency or the absence of infection.^36^ Blanket vaccination to ensure the target population is captured would be an alternative, but empirical data and modeling would be needed to assess the costs of vaccine wastage, and consideration given to the ethics of vaccinating individuals unable to derive direct benefit from vaccination.

Regarding prevention of disease versus prevention of infection vaccines, although several studies include vaccines with these different effect types, there tend to be other simultaneous changes in the vaccine profiles or targeting, such as age or infection status targeting, which confound the comparison of the impact of these two vaccine types. However, overall the studies suggest that prevention of disease vaccines tend to have a quicker and greater epidemiological impact than prevention of infection vaccines over the time horizons explored.

Theoretically, it is possible that there could be a genetic predisposition responsible for both ability to control *M.tb* latent infection and to respond to a vaccine. For a POD vaccine, this would become apparent in efficacy trials, as there would be no impact of such a vaccine on the disease endpoint. However, for POI vaccine studies with an infection endpoint, such a scenario could reduce infection rates, but have little impact on population level burden of disease as the vaccine may not be effective in those individuals most likely to progress to disease if infected. None of the POI vaccine models explored the potential scenario where vaccine efficacy is linked to likelihood to progress to disease, but this could be an interesting avenue for future research.

Although the relative impact of vaccine profiles to one another is informative for rational development of portfolio strategy, the absolute impact of such programs is important for understanding the potential role of such new technologies in achieving global targets and for advocacy for investment. Novel TB vaccines have the potential to provide an important contribution toward achieving the WHO 2035 and 2050 goals. Yet given the ambitious nature of the 2050 targets, even novel vaccines may require synergistic pairing with other interventions to achieve elimination in most modeled scenarios. Due to the sizeable global pool of latent infection, even a complete transmission block may not achieve elimination because of the continued burden from reactivation disease. Therefore, prevention of reactivation disease through vaccination or preventative treatment of latently infected individuals will be essential to elimination strategy.

Cost must also be considered when planning implementation of new TB vaccines, therefore health economic models will be essential. With the exception of one low-efficacy vaccine study and one low burden setting,^22,30^ the studies identified found new TB vaccines to be an overwhelmingly cost effective intervention. The results of threshold analyses are highly context dependent. However, in one analysis of low and middle income settings, vaccines targeted to adolescents and adults were shown to be cost effective as low as 20% vaccine efficacy and five years duration of protection, whereas infant vaccines required higher efficacies and longer durations to cross this threshold over the time horizons considered.^27^

An adapted tool was developed to assess quality and risk of bias of included studies for the purposes of this review to systematically assess reporting, methodological and risk of bias factors. The majority of included papers were scored as medium or high quality. The major gaps observed highlight for future studies the importance of thorough reporting and the conduct of comprehensive uncertainty and sensitivity analyses. It is hoped that this quality assessment tool will be of broad use in future systematic reviews assessing epidemiological models of other interventions and diseases.

The main limitation of this review was the conduct of independent sifting and data extraction by a single reviewer. The authors recognize that sifting by two independent reviewers remains the gold standard for systematic reviews.^6^ However, due to resource constraints this was not possible, but a very low threshold was applied for directing sifting queries to a second reviewer. In addition, the review was first conducted in 2014 and then repeated in 2016, therefore duplication of sifting by the same primary reviewer was expected to reduce the likelihood of missing relevant literature. It was found that the study quality assessment tool developed was not as well suited to assessment of analytical models as several domains were not applicable, leading to a higher likelihood of scoring ‘medium’ quality.

Several research gaps were identified in this analysis of the available literature. The lack of a clear explanation for the polarization of outcomes for pre-infection and post-infection vaccines is troubling, therefore a model to explore which key determinants within the model impact these outcomes would be an important addition to the literature. None of the models presented explicitly explored the potential impact of targeting vaccines to older adult or elderly populations. Such a model would be pertinent for a country such as China, which has high disease burden, an aging population, and has only been briefly explored in one sub-model in the literature. Future vaccines could be important in tackling multi-drug resistance disease through prevention of transmission or disease, yet drug resistance was not explored in any of the models identified. Few of the models included non-random social mixing patterns, and none considered the potential impact of evolving mixing patterns on the impact of vaccines. Some studies have explored the epidemiological impact of vaccine age targeting in sub-Saharan Africa; however, these models were either missing HIV structure, did not explore reduction of vaccine efficacy in HIV-infected individuals, only considered vaccination of uninfected populations, or were static models. Given HIV co-infection and high forces of *M.tb* infection are fundamental to the epidemic in many sub-Saharan African countries, there is a need for a comprehensive model incorporating these important elements.

## Conclusion

Mathematical modeling has been used to understand how the epidemiological impact of future vaccines could be altered by vaccine characteristics, vaccine age targeting, and epidemiological setting. It has also proved important for exploring the potential role of new vaccines for achieving the WHO 2050 goal of tuberculosis elimination. Such modeling should be integral to the development of future TB vaccines, informing rational decision making by cross-product bodies, academia, industry and policy makers for the development, investment and implementation of pipeline vaccines.

## Supplementary Material

Supplementary files

## Abbreviations

ART: Antiretroviral therapy
BCG: Bacillus Calmette-Guérin
CI: Confidence intervals
EPI: Expanded Programme on Immunization
*M.tb*: *Mycobacterium tuberculosis*
PICOS: Population, Intervention, Comparator, Outcome, Study design
POD: Prevention of Disease
POI: Prevention of Infection
POI&D: Prevention of Infection and Disease
P&PI: Pre- and post-infection
PRI: Pre-infection
PSI: Post-infection
SSA: sub-Saharan Africa
TB: Tuberculosis
VE: Vaccine efficacy
WHO: World Health Organization
