# Supplementary material for: Systematic review of mathematical models exploring the epidemiological impact of future TB vaccines
Source: Hum Vaccin Immunother. 2016 Jul 22;12(11):2813–32. doi: 10.1080/21645515.2016.1205769 (PMC5137531; doi:10.1080/21645515.2016.1205769)
Supplement: Supplementary files [file khvi-12-11-1205769-s001.zip › 2015HV0122R1-s02.docx]

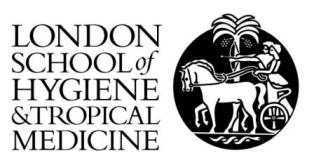

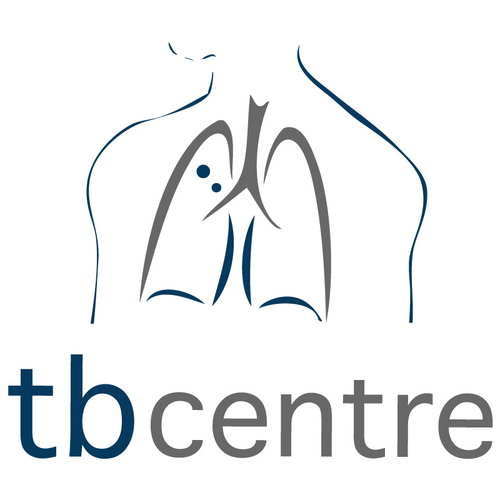

## Protocol: Systematic review of models exploring the epidemiological impact of novel TB vaccines

TB Modelling Group, LSHTM

Prepared by: Rebecca Harris

Document date: 15^th^ December 2015

Version: 1

**Senior supervisors: Dr Richard White**

**Project lead:** **Rebecca Harris**

**Project team: Dr Tom Sumner**

**Dr Gwen Knight**

Contact email: rebecca.harris@lshtm.ac.uk

**Literature search strategy**

**1.0 RESEARCH OBJECTIVES**

- 1. *Background*

With a strong pipeline of 15 novel tuberculosis (TB) vaccine candidates in clinical trials,^1^ there is a growing modelling literature exploring the potential impact of novel TB vaccines. Such models can be used to inform decision making with regards to the characteristics and target population for novel vaccines, to allow limited funding to be appropriately prioritised to ensure the greatest public health impact would be achieved when new vaccines come to market. To-date, the body of literature on this topic has not been summarised. The objective of this review is to narratively summarise the methodology and outcomes from modelling research exploring the epidemiological impact of pipeline or theoretical TB vaccines.

*1.2 Research Question*

What are the methods and estimated epidemiological impact of pipeline or theoretical human tuberculosis vaccines as estimated by mathematical modelling?

The PICOT framework for this research question is as follows:

Table 1: PICOT question

| Limit | Definition |
| --- | --- |
| Population | Humans, of any age in any country or globally |
| Intervention | Novel/theoretical/pipeline TB vaccines  *Not* BCG-only |
| Comparator | No intervention, currently available interventions (at current or scaled-up levels), or other theoretical interventions. |
| Outcome | Tuberculosis epidemiological impact (e.g. incidence, prevalence, mortality, number needed to vaccinate, cost effectiveness)  Not *Mycobacterium bovis* |
| Time | No limit |
| Methodology | Epidemiological mathematical models  *Not* within-host impact models  *Not* reviews/commentaries |

**2.0 METHODS**

*2.1 Search Strategy*

Three databases (Pubmed, Embase and WHO Global Index Medicus (GIM)) providing access to seven databases/indexes (Pubmed/Medline, Embase, African Index Medicus, LILACS, SEARO Index Medicus, WPRO Index Medicus, EMRO Index Medicus) will be searched. Searches will use free text and Mesh/Emtree/DeCS terms tailored by database for groups of terms covering tuberculosis, modelling and vaccines (table 2). Search terms are combined with Boolean OR within groups, and by Boolean AND between search term groups. The search in Pubmed will be run with filter “human”, and the WHO GIM search will be limited to regional databases to avoid duplication.

Table 2: Search terms by database

| Database | Search Term Group | | |
| --- | --- | --- | --- |
|  | Modelling | Tuberculosis | Vaccine |
| Pubmed | "Models, Theoretical"[Mesh]) OR "mathematical model*" | TB OR tuberculosis OR "Tuberculosis"[Mesh] | vaccin* OR immuniz* OR immunis* OR "Tuberculosis Vaccines"[Mesh] |
| Embase | ("mathematical model$".mp. OR mathematical model.mp. or mathematical model/) | (tuberculosis control/ or exp tuberculosis/ or Mycobacterium tuberculosis/ or tb.mp. or tuberculosis.mp.) | (exp vaccine/ or (vaccin$ or immunis$ or immuniz$).mp.) |
| WHO Global Index Medicus (regional databases) | ("computer models" OR "epidemiologic models" OR "mathematical models") | TB OR tuberculosis OR "tuberculosis" | Not required as very few hits with first two search terms |

Table 3: Application of PICOT through search limits and manual search criteria

| Limit | Definition | | Limit management |
| --- | --- | --- | --- |
| Population |  | Humans, of any age in any country or globally | “Human” search limit (pubmed only) and manual search criterion |
| Intervention |  | Novel/theoretical/pipeline TB vaccines  *Not* BCG-only | Vaccine search terms and manual search criteria |
| Comparator |  | No intervention, currently available interventions (at current or scaled-up levels), or other theoretical interventions. | Manual search criteria |
| Outcome |  | Tuberculosis epidemiological impact (e.g. incidence, prevalence, mortality, number needed to vaccinate, cost effectiveness)  Not *Mycobacterium bovis* | TB search terms and manual search criteria |
| Time |  | No limit | No limit applied |
| Methodology |  | Epidemiological mathematical models  *Not* within-host impact models  *Not* reviews/commentaries | Modelling search terms manual search criteria |

*2.2 Selection of studies and data extraction*

The database searching, sifting and data extraction will be conducted in by a single reviewer (RCH). A three-stage sifting process will be employed to screen publications first at title, abstract, then at full text level for eligibility for inclusion using the below-details inclusion and exclusion criteria. Any uncertainties in inclusion will be decided through discussion with a second reviewer (RW). Reference lists of included studies will be hand searched for studies meeting the inclusion criteria. Onward citation searching will be conducted for all included articles.

*Inclusion Criteria*

- Mathematical model
- Systematic review of models of novel TB vaccines, or commentary adding to the analyses/interpretation of models reported elsewhere
- Intervention is novel/future/hypothetical vaccine against tuberculosis or of an unspecified novel TB intervention with characteristics in-line with a vaccine
- Reported outcomes are of epidemiological impact of vaccine (e.g. incidence, prevalence, mortality, number needed to vaccinate, cost effectiveness)

*Exclusion Criteria*

- Within-host/immunological vaccine impact models
- Review or commentary not adding to existing body of knowledge
- TB epidemiological models not reporting impact of vaccine
- TB epidemiological models reporting only interventions other than vaccines
- Model only reporting on impact of BCG with known/fixed efficacy
- Disease or infection caused by *Mycobacterium bovis* or other non-*Mycobacterium tuberculosis* strain.

Data will be extracted from those papers selected for final inclusion using a standardised Microsoft Excel® database. Extracted data will include study objectives, model methods (e.g. model structure, mixing patterns, model assumptions, parameter data sources), intervention characteristics (e.g. vaccine efficacy, duration of protection, waning of protection, vaccine target population and schedule), epidemiological outcomes, sensitivity analyses, and model limitations.

**3.0 QUALITY ASSESSMENT AND DATA SYNTHESIS**

## 3.1 Quality Assessment

Few validated tools exist for the assessment of quality of epidemiological modelling studies. Fone *et al.* (2003) have proposed and tested an adapted version of the Weightman *et al.* (2000) tool for the critical appraisal of the quality of modelling studies in health care.^2,3^ And more recently Caro *et al*. have detailed the criteria for good research practice in modelling research.^4^ In this review, the Fone *et al.* tool will be used as a basis for quality assessment, but updated based upon the Caro *et al.* report to ensure clarity and coverage of certain criteria.^2,4^ Based upon extracted data, each included article will be critically appraised for quality using a piloted version of this extraction form.

## 3.2 Data Synthesis

Extracted data will be synthesised using a narrative approach and will focus on the modelling methods used, estimates of epidemiological impact, and identifying evidence gaps or limitations.

## 3.3 Dissemination

The review manuscript will be submitted for publication in a peer reviewed journal.

*4.0 References*

1. World Health Organization. Global Tuberculosis Report 2015. Geneva, Switzerland, 2015.

2. Fone D, Hollinghurst S, Temple M, et al. Systematic review of the use and value of computer simulation modelling in population health and health care delivery. *Journal of public health medicine* 2003; **25**(4): 325-35.

3. Weightman A, Barker J, Lancaster J. Health Evidence Bulletins Wales Project Methodology 3. . Cardiff: Department of Information Services: UWCM, 2000.

4. Caro JJ, Briggs AH, Siebert U, Kuntz KM. Modeling good research practices--overview: a report of the ISPOR-SMDM Modeling Good Research Practices Task Force--1. *Value in health : the journal of the International Society for Pharmacoeconomics and Outcomes Research* 2012; **15**(6): 796-803.
