# Supplementary material for: Systematic review of mathematical models exploring the epidemiological impact of future TB vaccines
Source: Hum Vaccin Immunother. 2016 Jul 22;12(11):2813–32. doi: 10.1080/21645515.2016.1205769 (PMC5137531; doi:10.1080/21645515.2016.1205769)
Supplement: Supplementary files [file khvi-12-11-1205769-s001.zip › 2015HV0122R1-s01.docx]

**Supplementary Materials**

Table S1: PICOS framework for the research question

| Limit | Definition | Limit management |
| --- | --- | --- |
| Population | Humans, any age any country | Search limit |
| Intervention | Novel/theoretical/pipeline TB vaccines  *Not* single efficacy BCG-only | Search terms and exclusion criteria |
| Comparator | No intervention, currently available interventions (at current or scaled-up levels), or other theoretical interventions. | No limit applied |
| Outcome | Tuberculosis epidemiological impact (incidence, prevalence, mortality, number needed to vaccinate, cost effectiveness)  Not *Mycobacterium bovis*  *Not* within-host impact models | Inclusion/exclusion criteria |
| Time | No limit | No limit applied |
| Study Design | Epidemiological mathematical models  *Not* reviews/commentaries | Search terms and Inclusion/exclusion criteria |

Table S2: Full search strategies for each database

| Database | Search Term Group | | |
| --- | --- | --- | --- |
|  | Modelling | Tuberculosis | Vaccine |
| Pubmed | "Models, Theoretical"[Mesh]) OR "mathematical model*" | TB OR tuberculosis OR "Tuberculosis"[Mesh] | vaccin* OR immuniz* OR immunis* OR "Tuberculosis Vaccines"[Mesh] |
| Embase | ("mathematical model$".mp. OR mathematical model.mp. or mathematical model/) | (tuberculosis control/ or exp tuberculosis/ or Mycobacterium tuberculosis/ or tb.mp. or tuberculosis.mp.) | (exp vaccine/ or (vaccin$ or immunis$ or immuniz$).mp.) |
| WHO Global Health Library | ("computer models" OR "epidemiologic models" OR "mathematical models") | TB OR tuberculosis OR "tuberculosis" | Not required as very few hits with first two search terms |

Search terms were combined with Boolean OR within groups, and by Boolean AND between search term groups.

S3: Risk of bias tool for assessment of epidemiological modelling studies

|  | **Criterion (adapted from Fone *et al.* and Caro *et al.)*** | | **Considerations (adapted from Fone et al. and Caro et al.)** | **Score considerations (0, poor to 2, good)** |  |
| --- | --- | --- | --- | --- | --- |
| 1 | **Are the aims and objectives clear?** | | Are the research questions and modelling objectives clearly defined? | 0 Not stated  1 Stated but vague  2 Stated and focussed | Definitions: max 8 points |
| 2 | **Is the setting and population clearly defined?** | | Does the paper clearly state the setting (e.g. geographical location, high/low TB burden)? | 0 Not stated  1 Stated but vague or details missing  2 Stated and focussed |  |
|  |  |  | In health economics models, has the perspective been stated? |  |  |
|  |  |  | Does the paper clearly state the modelled population? (e.g. patient or population group characteristics) |  |  |
|  |  |  | Have sub-populations necessary for the research question and setting been modelled? |  |  |
| 3 | **Are the intervention and comparators adequately defined?** | | Does the paper clearly state the population(s) targeted for vaccination? | 0 Not stated or very unclear  1 Stated but details missing  2 Stated and all necessary details stated |  |
|  |  |  | Does the paper clearly define the vaccine characteristics (e.g. vaccine efficacy, duration of protection, number of doses, waning, timing)? |  |  |
|  |  |  | If there is a comparator (no vaccine, baseline or alternative intervention scenario), is it clearly defined? |  |  |
| 4 | **Are the outcome measures defined and answer the research question?** | | Does the paper clearly define the outcomes of interest? | 0 Not stated, very unclear or not suited to research question  1 Stated but details missing or not directly aligned with research question  2 Stated, all necessary details stated, and aligned with research question |  |
|  |  |  | Do the outcomes correspond to the research question? |  |  |
| 5 | **Are the model structure and time horizon clearly described and appropriate for the research question?** | | Is the model structure clearly reported and appropriate for the research question? | 0 Not appropriate model structure, or poor/no description of model  1 Incomplete description, and/or appropriate in part for research question  2 Complete and reproducible, appropriate structure and time horizon | Model methods: max 4 points |
|  |  |  | Does the model reflect current knowledge of disease natural history? |  |  |
|  |  |  | Is the time horizon and time step of the model clearly stated and appropriate to the research question (i.e. is it long enough to capture health effects)? |  |  |
| 6 | **Are the modelling methods appropriate for the research question and adequately described?** | | Were the modelling methods clearly described, and suited to the research question? | 0 Not appropriate model structure, or poor/no description of methods  1 Incomplete description, and/or appropriate in part for research question  2 Complete and reproducible, appropriate method |  |
| 7 | **Are the parameters, ranges and data sources specified?** | | Are all parameters and their ranges reported? | 0 Poorly reported  1 Some information missing  2 Complete reporting of parameters, ranges and data sources | Model inputs: max 6 points |
|  |  |  | Are the data sources for parameters reported? |  |  |
| 8 | **Are any assumptions explicit and justified?** | | Are all assumptions explicit and justified? | 0 Not reported  1 Explicit  2 Explicit and justified |  |
| 9 | **Is the quality of data considered and is uncertainty explored through uncertainty and/or sensitivity analyses?** | | Are data limitations discussed? Are any of the sources known to the reviewer to be inappropriate (e.g. do not match the parameter, are outdated, or known to be poor quality)? | 0 No sources or uncertainty  1 Partially addressed, and/or some data inappropriate  2 Fully addressed |  |
|  |  |  | Is uncertainty in model structure, parameters and/or assumptions explored through uncertainty and/or sensitivity analyses? |  |  |
| 10 | **Is the method of fitting described and suitable?** | | Is the method of fitting/calibrating the model clearly described? | 0 Not done, unsuitable method or poor/no description  1 Incomplete description or method not optimal  2 Complete description and suitable methods | Fitting/ validation: max 4 points |
|  |  |  | Is the method of model fitting/calibration suitable? |  |  |
| 11 | **Has the model been validated?** | | Has an assessment of validity of the results been made by comparing across one or more different model structures, or against a validation data set? | 0 Not considered  1 States criteria for validation  2 Validation undertaken |  |
| 12 | **Have the results been clearly and completely presented, with a range of uncertainty?** | | Have the outcome values and their uncertainty ranges for each intervention/scenario been reported? | 0 Not reported, very unclear or not suited to research question  1 Stated, but ranges or planned sensitivity analyses missing and/or not directly aligned with research question  2 Values and ranges and planned sensitivity analyses reported and aligned with research question. | Results: max 4 points |
|  |  |  | Do the results match the objectives? |  |  |
|  |  |  | Are sensitivity analyses clearly reported? |  |  |
| 13 | **Are the results appropriately interpreted and discussed in context?** | | Does the discussion reflect a fair and balanced interpretation of the results? | 0 No/poor discussion  1 Some discussion but key points, limitations or context missed  2 Full discussion of key points in context, generalisability considered, limitations discussed |  |
|  |  |  | Are the results of the study discussed in context and is generalisability considered? |  |  |
|  |  |  | Are possible biases and limitations discussed? |  |  |
| 14 | **Are the funding source and conflicts of interest reported?** | | Is the funding and the role of the funder clearly stated? | 0 No statement of funding or conflicts  1 Funding or conflicts reported  2 Funding and conflict statement | Conflicts:  Max 2 points |
|  |  |  | Is there a conflict of interest statement? |  |  |
|  |  | |  |  | |
| Overall Scoring: Max 28 points | | |  |  | |
| Very high | | >22 |  |  | |
| High | | 19-22 |  |  | |
| Medium | | .14-18 |  |  | |
| Low | | <14 |  |  | |

Table S4: PRISMA 2009 Checklist

| **Section/topic** | **#** | **Checklist item** | **Reported on page #** |
| --- | --- | --- | --- |
| **TITLE** | | |  |
| Title | 1 | Identify the report as a systematic review, meta-analysis, or both. | 1 |
| **ABSTRACT** | | |  |
| Structured summary | 2 | Provide a structured summary including, as applicable: background; objectives; data sources; study eligibility criteria, participants, and interventions; study appraisal and synthesis methods; results; limitations; conclusions and implications of key findings; systematic review registration number. | 4 |
| **INTRODUCTION** | | |  |
| Rationale | 3 | Describe the rationale for the review in the context of what is already known. | 5-7 |
| Objectives | 4 | Provide an explicit statement of questions being addressed with reference to participants, interventions, comparisons, outcomes, and study design (PICOS). | 6, Table S1 |
| **METHODS** | | |  |
| Protocol and registration | 5 | Indicate if a review protocol exists, if and where it can be accessed (e.g., Web address), and, if available, provide registration information including registration number. | 9, supp. appendix C |
| Eligibility criteria | 6 | Specify study characteristics (e.g., PICOS, length of follow-up) and report characteristics (e.g., years considered, language, publication status) used as criteria for eligibility, giving rationale. | 6, 8-9, Table S1 |
| Information sources | 7 | Describe all information sources (e.g., databases with dates of coverage, contact with study authors to identify additional studies) in the search and date last searched. | 8-9 |
| Search | 8 | Present full electronic search strategy for at least one database, including any limits used, such that it could be repeated. | Table S2 |
| Study selection | 9 | State the process for selecting studies (i.e., screening, eligibility, included in systematic review, and, if applicable, included in the meta-analysis). | 8-9 |
| Data collection process | 10 | Describe method of data extraction from reports (e.g., piloted forms, independently, in duplicate) and any processes for obtaining and confirming data from investigators. | 9 |
| Data items | 11 | List and define all variables for which data were sought (e.g., PICOS, funding sources) and any assumptions and simplifications made. | - |
| Risk of bias in individual studies | 12 | Describe methods used for assessing risk of bias of individual studies (including specification of whether this was done at the study or outcome level), and how this information is to be used in any data synthesis. | 9-10, Table S3 |
| Summary measures | 13 | State the principal summary measures (e.g., risk ratio, difference in means). | 8 |
| Synthesis of results | 14 | Describe the methods of handling data and combining results of studies, if done, including measures of consistency (e.g., I^2^) for each meta-analysis. | n/a |

Page 1 of 2

| **Section/topic** | **#** | **Checklist item** | **Reported on page #** |
| --- | --- | --- | --- |
| Risk of bias across studies | 15 | Specify any assessment of risk of bias that may affect the cumulative evidence (e.g., publication bias, selective reporting within studies). | n/a |
| Additional analyses | 16 | Describe methods of additional analyses (e.g., sensitivity or subgroup analyses, meta-regression), if done, indicating which were pre-specified. | n/a |
| **RESULTS** | | |  |
| Study selection | 17 | Give numbers of studies screened, assessed for eligibility, and included in the review, with reasons for exclusions at each stage, ideally with a flow diagram. | 11, Figure 1 |
| Study characteristics | 18 | For each study, present characteristics for which data were extracted (e.g., study size, PICOS, follow-up period) and provide the citations. | 11-15, Table 3 |
| Risk of bias within studies | 19 | Present data on risk of bias of each study and, if available, any outcome level assessment (see item 12). | 26,Table 4 |
| Results of individual studies | 20 | For all outcomes considered (benefits or harms), present, for each study: (a) simple summary data for each intervention group (b) effect estimates and confidence intervals, ideally with a forest plot. | 15-25 |
| Synthesis of results | 21 | Present results of each meta-analysis done, including confidence intervals and measures of consistency. | n/a |
| Risk of bias across studies | 22 | Present results of any assessment of risk of bias across studies (see Item 15). | n/a |
| Additional analysis | 23 | Give results of additional analyses, if done (e.g., sensitivity or subgroup analyses, meta-regression [see Item 16]). | n/a |
| **DISCUSSION** | | |  |
| Summary of evidence | 24 | Summarize the main findings including the strength of evidence for each main outcome; consider their relevance to key groups (e.g., healthcare providers, users, and policy makers). | 26-32 |
| Limitations | 25 | Discuss limitations at study and outcome level (e.g., risk of bias), and at review-level (e.g., incomplete retrieval of identified research, reporting bias). | 32-33 |
| Conclusions | 26 | Provide a general interpretation of the results in the context of other evidence, and implications for future research. | 33-34 |
| **FUNDING** | | |  |
| Funding | 27 | Describe sources of funding for the systematic review and other support (e.g., supply of data); role of funders for the systematic review. | 2 |

*From:*  Moher D, Liberati A, Tetzlaff J, Altman DG, The PRISMA Group (2009). Preferred Reporting Items for Systematic Reviews and Meta-Analyses: The PRISMA Statement. PLoS Med 6(6): e1000097. doi:10.1371/journal.pmed1000097

**Supplementary appendix A: Model structures and fitting methods**

Model structures have evolved based on the growing body of knowledge about TB natural history and epidemiology, in addition to being tailored to each research question. The majority of models include the susceptible (S), latent (L), active disease (I) and recovered (R) natural history states. Some models include latent fast and slow progressing states,^1-4^ though many models bypass the ‘latent fast’ state to transition directly to either stable latent infection or active disease. ^5, 6^ Other features included in some models are: treatment status,^1, 6^ variable infectiousness of active disease or disease type,^7-11^ different infective strains,^12^ vaccine waning,^3^ and stratification for age, HIV,^6, 9, 13, 14^ community structure,^15^ and vaccination. Markov models identified also included other health states such as re-infected,^16^ miliary TB,^16^ TB meningitis^16^, diagnosis,^13^ treatment status,^13, 14^ and drug resistance status^13, 14^ health states.

Fitting methods reported included Sobol sequence sampling and approximate Bayesian computation,^9^ Downhill simplex method,^1^ maximum likelihood,^17^ or adjustment of parameters to achieve required equilibrium values using Berkley Madonna “curve fit” routine,^7, 8^ generalised reduced gradient non linear engine of solver in Excel or manually.^6-8^ Fitting methods were unclear or not reported in six of the models,^2-4, 10-12^ and illustrative examples, Markov or analytical models did not require fitting.^5, 13-16, 18-22^

**Supplementary appendix B: Additional summary of quality scoring**

Study aims and outcome measures tended to be well defined, but there was often missing information with regards to the modelled population, vaccine characteristics, time horizon, on occasion model structure and parameter ranges and references. The model structure and methods were generally suited to the research question, but some models had out-dated natural history structures or inappropriate time horizons. Overall, the scoring for fitting methods was relatively low, as several studies lacked or were unclear in their reporting, plus this is not a relevant criterion for theoretical papers. Model assumptions were generally stated and justified. Studies were incredibly variable with respect to the completeness of conduct and reporting of uncertainty ranges and sensitivity analyses. Points were lost in reporting of results due to poor reporting of uncertainty and sensitivity analyses, but overall results were appropriately interpreted and discussed in context. Only one model had been validated, and most papers were missing a conflict of interest and/or funding statement.

**References**

1. Abu-Raddad LJ, Sabatelli L, Achterberg JT, Sugimoto JD, Longini IM, Jr., Dye C, Halloran ME. Epidemiological benefits of more-effective tuberculosis vaccines, drugs, and diagnostics. Proceedings of the National Academy of Sciences of the United States of America 2009; 106:13980-5.

2. Bhunu CP, Garira W, Mukandavire Z, Magombedze G. Modelling the effects of pre-exposure and post-exposure vaccines in tuberculosis control. Journal of theoretical biology 2008; 254:633-49.

3. Lietman T, Blower SM. Potential impact of tuberculosis vaccines as epidemic control agents. Clinical infectious diseases : an official publication of the Infectious Diseases Society of America 2000; 30 Suppl 3:S316-22.

4. Ziv E, Daley CL, Blower S. Potential public health impact of new tuberculosis vaccines. Emerging infectious diseases 2004; 10:1529-35.

5. Gomes MG, Franco AO, Gomes MC, Medley GF. The reinfection threshold promotes variability in tuberculosis epidemiology and vaccine efficacy. Proceedings Biological sciences / The Royal Society 2004; 271:617-23.

6. Murray CJ, Salomon JA. Modeling the impact of global tuberculosis control strategies. Proceedings of the National Academy of Sciences of the United States of America 1998; 95:13881-6.

7. Dye C, Williams BG. Eliminating human tuberculosis in the twenty-first century. Journal of the Royal Society, Interface / the Royal Society 2008; 5:653-62.

8. Dye C, Glaziou P, Floyd K, Raviglione M. Prospects for tuberculosis elimination. Annual review of public health 2013; 34:271-86.

9. Knight GM, Griffiths UK, Sumner T, Laurence YV, Gheorghe A, Vassall A, Glaziou P, White RG. Impact and cost-effectiveness of new tuberculosis vaccines in low- and middle-income countries. Proceedings of the National Academy of Sciences of the United States of America 2014; 111:15520-5.

10. Dye C. Tuberculosis 2000-2010: control, but not elimination [The Comstock Lecture]. The International Journal of Tuberculosis and Lung Disease 2000; 4:S146-S52.

11. ReVelle CS, Lynn WR, Feldmann F. Mathematical models for the economic allocation of tuberculosis control activities in developing nations. American review of respiratory disease 1967; 96:893-909.

12. Cohen T, Colijn C, Murray M. Modeling the effects of strain diversity and mechanisms of strain competition on the potential performance of new tuberculosis vaccines. Proceedings of the National Academy of Sciences of the United States of America 2008; 105:16302-7.

13. Tseng CL, Oxlade O, Menzies D, Aspler A, Schwartzman K. Cost-effectiveness of novel vaccines for tuberculosis control: a decision analysis study. BMC public health 2011; 11:55.

14. Ditkowsky JB, Schwartzman K. Potential cost-effectiveness of a new infant tuberculosis vaccine in South Africa--implications for clinical trials: a decision analysis. PloS one 2014; 9:e83526.

15. Pienaar E, Fluitt AM, Whitney SE, Freifeld AG, Viljoen HJ. A model of tuberculosis transmission and intervention strategies in an urban residential area. Computational biology and chemistry 2010; 34:86-96.

16. Channing L, Sinanovic E. Modelling the cost-effectiveness of a new infant vaccine to prevent tuberculosis disease in children in South Africa. Cost Effectiveness and Resource Allocation 2014; 12:1-9.

17. Dye C. Making wider use of the world's most widely used vaccine: Bacille Calmette-Guerin revaccination reconsidered. Journal of the Royal Society, Interface / the Royal Society 2013; 10:20130365.

18. Castillo-Chavez C, Feng Z. Global stability of an age-structure model for TB and its applications to optimal vaccination strategies. Mathematical biosciences 1998; 151:135-54.

19. Gabriela MGM, Rodrigues P, Hilker FM, Mantilla-Beniers NB, Muehlen M, Cristina Paulo A, Medley GF. Implications of partial immunity on the prospects for tuberculosis control by post-exposure interventions. Journal of theoretical biology 2007; 248:608-17.

20. Hawn TR, Day TA, Scriba TJ, Hatherill M, Hanekom WA, Evans TG, Churchyard GJ, Kublin JG, Bekker LG, Self SG. Tuberculosis vaccines and prevention of infection. Microbiology and molecular biology reviews : MMBR 2014; 78:650-71.

21. Rahman M, Sekimoto M, Takamatsu I, Hira K, Shimbo T, Toyoshima K, Fukui T. Economic evaluation of universal BCG vaccination of Japanese infants. International journal of epidemiology 2001; 30:380-5.

22. Rodrigues P, Margheri A, Rebelo C, Gomes MG. Heterogeneity in susceptibility to infection can explain high reinfection rates. Journal of theoretical biology 2009; 259:280-90.
